# Supplementary material for: Uttarakhand State Earthquake Early Warning System: A Case Study of the Himalayan Environment
Source: Sensors (Basel). 2024 May 21;24(11):3272. doi: 10.3390/s24113272 (PMC11174874; doi:10.3390/s24113272)
Supplement: Supplementary file 1 [file sensors-24-03272-s001.zip › sensors-2895203-supplementary.pdf]

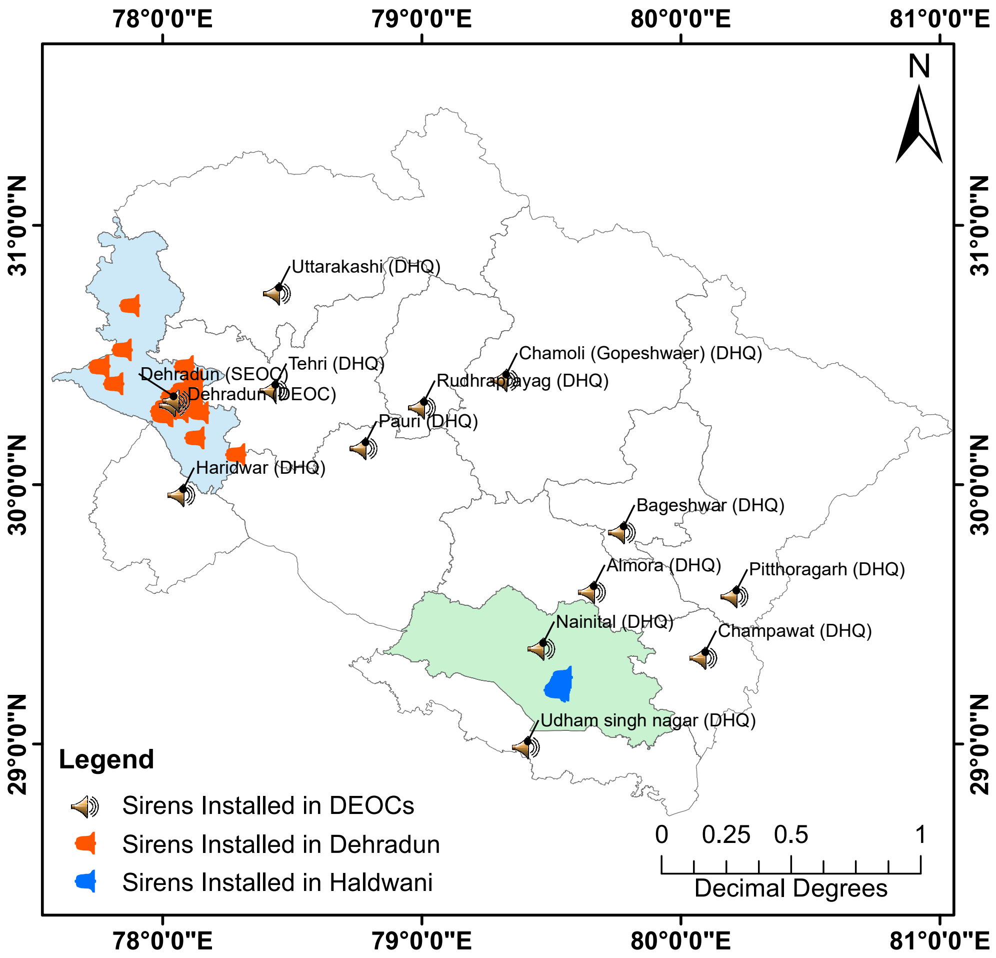

Figure S1. Location of installed siren units in Uttarakhand.

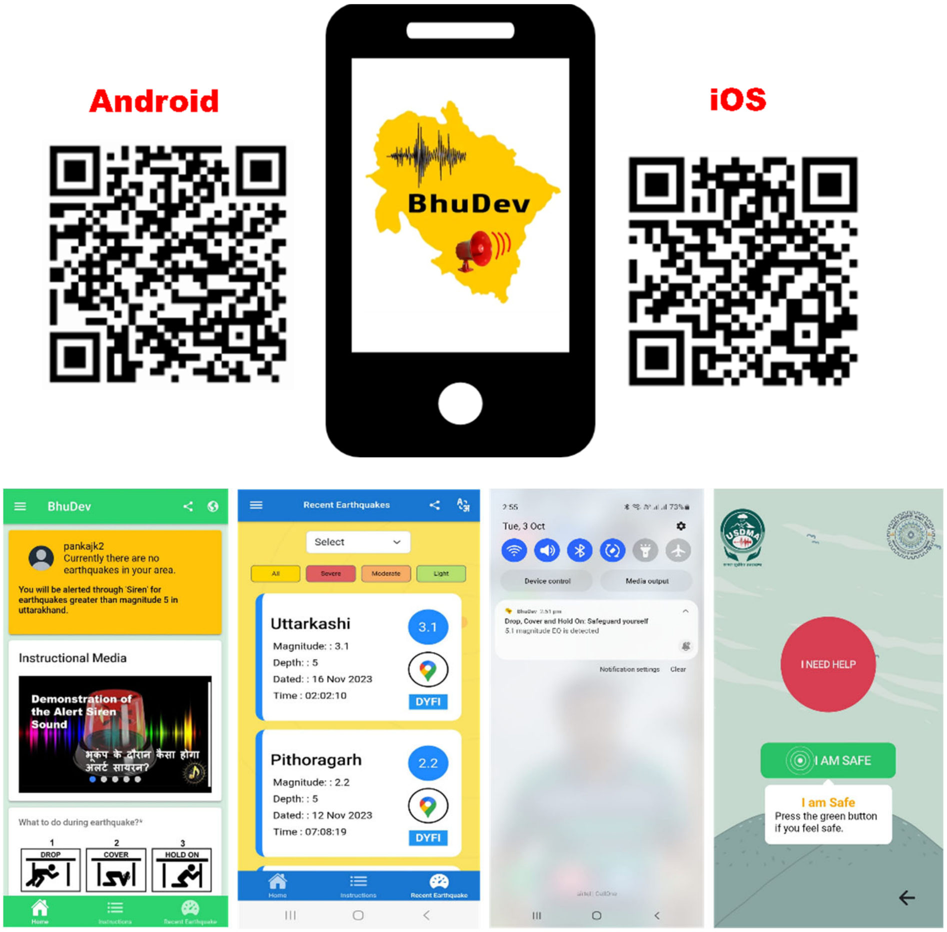

Figure S2. The icon and QR codes for the Bhukamp Disaster Early Vigilantè (BhuDEV) app. A few pages of the app are also shown.
